# Medical Nutrition Therapy in Diabetes Mellitus: New Insights to an Old Problem

**DOI:** 10.3390/nu14030698

**Published:** 2022-02-07

**Authors:** Maria G. Grammatikopoulou, Dimitrios G. Goulis

**Affiliations:** 1 Department of Nutritional Sciences and Dietetics, Faculty of Health Sciences, Alexander Campus, International Hellenic University, GR-57400 Thessaloniki, Greece; mariagram@auth.gr; 2 Unit of Reproductive Endocrinology, 1st Department of Obstetrics and Gynecology, Medical School, Aristotle University of Thessaloniki, Papageorgiou General Hospital, GR-56429 Thessaloniki, Greece

The management of all types of diabetes mellitus (DM) has transformed during the past decade. Apart from oral and injectable antidiabetic agents, a turn towards an effective application of medical nutrition therapy (MNT) for diabetes is evident. Optimizing adherence to MNT, preventing DM through MNT, finding the optimal MNT, and applying MNT for DM comorbidities are old problems; the present Special Issue of *Nutrients* provides new research findings and adds novel pieces to the puzzle regarding the effective application of MNT to patients with DM.

MNT is an integral part of treatment in prediabetes, gestational diabetes mellitus (GDM), and type 1 and type 2 diabetes mellitus (T1DM, T2DM) [1,2]. However, recent research has revealed that patients often fail to adhere to the recommendations [3]. Overall, only 3.7% of patients with T2DM in Greece had received nutrition education from a registered nutritionist-dietitian. Patients on oral antidiabetic agents were more adherent compared to those on insulin therapy, and men were more compliant compared to women [3]. These findings highlight the need for improved patient education concerning the optimal MNT. Indeed, according to a randomized controlled trial (RCT) conducted in Taiwan [4], an intensive and comprehensive health-coaching intervention for 6 months improved diet quality and glycemic control (assessed as HbA_1c_ concentrations) among patients with T2DM. Thus, the delivery of nutrition education should be considered a priority and an investment for improved outcomes among patients with DM.

More recently, mobile health (mHealth) and electronic health (eHealth) technologies have been employed to improve the delivery of DM-self management and patient education. A systematic review revealed that digital tools effectively support self-management related to diet, health behavior, and treatment adherence in women with GDM [5]. Furthermore, participants receiving mHealth- and eHealth-delivered interventions demonstrated a low attrition rate and a correspondingly high retention rate, suggesting that digital tools might increase treatment adherence among women with GDM [5].

The core of GDM prevention stems from lifestyle modifications and, in particular, adherence to healthy dietary patterns and appropriate physical activity levels [6]. According to a comprehensive review by Mierzyński [6], dietary modifications, including probiotics, fiber, vitamin D, and oral nutrient supplementation (ONS), can reduce the risk for developing GDM when paired with certain dietary patterns, such as the low-carbohydrate diet or the Mediterranean diet (MD).

In this manner, the St. Carlos GDM prevention study was an ambitious, innovative RCT that evaluated the incidence of GDM with two different dietary models, an MD supplemented with extra virgin olive oil (EVOO) and pistachios, or a standard diet with reduced fat intake [7]. The analysis showed that the incidence of GDM was lower in the MD arm compared to in the arm prescribed the standard diet. In a consecutive, pragmatic study [8], the authors opted to provide the same intervention recommendations (MD with EVOO and pistachios) through routine clinical practice to a real-world (RW) group of normoglycemic, pregnant women in their 12–14th week of gestation. The results were compared to those previously obtained from the two RCT arms. Interestingly, RW women exhibited a low incidence of GDM, similar to that of women receiving the MD intervention as part of the RCT. In parallel, the rates of urinary tract infections, emergency cesarean sections, and perineal trauma were lower in the RCT-MD intervention arm and in RW women [8]. The findings suggest that pregnant women can adopt MD interventions either as part of an RCT or as recommendations provided in everyday clinical practice. Furthermore, irrespective of the setting (RCT or RW), the preventive effect of MD for the development of GDM is similar.

DM is a multifactorial disease with different phenotypes and great variability in the individual response to MNT, indicating that genetic traits might interplay with treatment response. Gkouskou et al. [9] highlighted the importance of nutrigenetic applications for precision MNT in DM to unravel this hypothesis. The authors suggest that improved glycemic control can be achieved by prescribing MNT based on the MD, according to the genetic information of each patient in a personalized manner. Furthermore, they provide evidence supporting this concept by presenting a patient with T2DM who achieved acute improvements in glycemic control when they adhered to a personalized, genetically guided MD based on single nucleotide polymorphisms (SNPs) and genetic risk scores (GRS) data [9]. The prescription of genetically guided MNT appears to be the only way to deliver personalized MNT, extending beyond the provision of universal recommendations for all patients [10].

The delivery of MD-based MNT for T2DM was assessed by Al-Aubaidy et al. [11]. In a post hoc analysis of a cross-over RCT [12], in addition to improvements in the glycemic control, adherence to a traditional Cretan MD for 12 weeks increased plasma citrus bioflavonoids (naringin, hesperitin, and hesperidin) and reduced pro-inflammatory cytokines and oxidative stress markers (interleukin-6 (IL-6) and 8-hydroxy-2′-deoxyguanosine (8-OHdG)) compared to their usual diet [11]. These findings suggest an inti-inflammatory response following an MD.

Light was also shed on gray areas regarding MNT in women with GDM. According to the clinical practice guidelines for GDM and the relevant primary research, recommendations regarding the optimal estimated energy requirements (EER) for women with GDM are conflicting [1]. Along these lines, Tsirou et al. [13] conducted a non-randomized clinical study comparing two different energy regimes, a low-energy diet (LED) and a very low-energy diet (VLED), among women with a GDM diagnosis. The results revealed that gestational weight gain (GWG) was lower in the VLED; however, no differences were noted regarding the composite maternal–fetal score and type of delivery, infant birth weight, prematurity and Apgar score, maternal depression, or insulin use. Overall, this study suggests that adherence to an LED or VLED results in similar maternal, infant, and obstetrical outcomes; thus, there is no need to prescribe a more restrictive diet to women with GDM [13].

Although adherence to a healthy diet is an integral component of DM treatment that is tightly linked to glycemic control, patients with DM often report feeling excessively preoccupied with their diet, with disordered eating patterns being quite common. Orthorexia Nervosa (ON) is an atypical eating disorder described by an exaggerated, unhealthy obsession with healthy eating [14]. A comprehensive systematic review [15] revealed that patients with DM often exhibit ON tendencies, although research is still limited concerning the etiology of ON and the distinct characteristics of patients with a dual ON–DM diagnosis. Since disordered eating is associated with restrictive diets, inadequate micronutrient intake, and impaired glycemic control, the prompt diagnosis of patients with DM and ON becomes of great importance. Furthermore, all of the clinicians and health professionals employed in DM treatment should be aware of the problem and should evaluate ON tendencies in patients presenting signs of an unhealthy obsession with healthy eating [15].

Finally, given that exercise is an important part of DM treatment, Tsiroukidou et al. [16] evaluated the prognostic value of the hematological parameters of childhood obesity as potential predictors of cardiorespiratory fitness (CRF via VO_2max_) in children and adolescents with obesity and who were at risk for developing diabetes. Among children at high risk for T2DM, VO_2max_ could be predicted from leptin and fibrinogen, offering an alternative against invasive methods. Furthermore, the authors review the evidence regarding exercise and diet interventions for improving CRF among children and adolescents at risk for DM, thus proposing therapeutic options for its prevention [16].

As DM is a global health issue, research on several aspects of MNT in DM is perpetually evolving, adding new insights, and shedding light on novel therapeutic options. In this Special Issue of *Nutrients*, prominent experts have critically appraised the literature and have provided primary research data for MNT use in patients with DM.

## Data Availability

Not applicable.
